# Association between carotid intima media thickness and small dense low-density lipoprotein cholesterol in acute ischaemic stroke

**DOI:** 10.1186/s12944-020-01353-0

**Published:** 2020-07-28

**Authors:** Peiyang Zhou, Yan Shen, Lingyun Wang, Zhihua Cao, Wenmin Feng, Jincheng Liu, Lijun Wang, Peng Meng, Jinbo Yang, Wang-Yang Xu, Ping Gao

**Affiliations:** 1 Department of Neurology, Xiangyang No. 1 People’s Hospital, Hubei University of Medicine, No. 15 Jiefang Road, Fancheng District, Xiangyang, 441000 People’s Republic of China; 2 Research Center for Experimental Medicine, Ruijin Hospital, Affiliated to Shanghai Jiao Tong University School of Medicine, Shanghai, 200025 China; 3 Biotecan Medical Diagnostics Co.,Ltd., Zhangjiang Center for Translational Medicine, Shanghai, 201204 China; 4 Shanghai Zhangjiang Institute of Medical Innovation, Shanghai, 201204 China; 5 Department of Clinical Laboratory, Xiangyang No. 1 People’s Hospital, Hubei University of Medicine, Xiangyang, 441000 China

**Keywords:** Intima media thickness, Small dense low-density lipoprotein cholesterol, Independent risk factor, Carotid plaque, Atherosclerosis, Acute ischaemic stroke risk, Logistic regression model

## Abstract

**Background:**

Intima-media thickness (IMT) and small dense low-density lipoprotein cholesterol (sdLDL-C) have been reported to be related to atherosclerosis and stroke. This study is trying to explore the association between IMT and sdLDL-C in Chinese acute ischaemic stroke (AIS) subjects.

**Methods:**

This study enrolled total 368 consecutive AIS patients and 165 non-AIS controls from November 2016 to February 2019. Mean IMT and carotid plaques were measured by using carotid ultrasonography method. Blood glucose and lipid parameters were measured by using an automatic biochemical instrument. SdLDL-C was detected by using the Lipoprint LDL system. IMT > 1.0 mm was defined as increased IMT. Plaque stability based on the nature of the echo was determined by ultrasound examination. Risk factors for IMT were identified by using multivariate logistic regression analysis. A logistic regression model was established to predict AIS risk. Python software (Version 3.6) was used for the statistical analysis of all data.

**Results:**

The carotid IMT, proportion of plaques, and the sdLDL-C, triglycerides (TG) and glucose levels were obviously higher in AIS patients than those in controls. SdLDL-C level in the IMT thickening group was higher than that in the normal IMT group. SdLDL-C and total cholesterol (TC) were risk factors for IMT, while sdLDL-C was an independent risk factor. The IMT value of the unstable plaque group was markedly higher than that of the stable plaque group. The predictive value of IMT for AIS was better than that of low-density lipoprotein cholesterol (LDL-C) and non-high-density lipoprotein cholesterol (non-HDL-C) but not as good as that of sdLDL-C. A logistic regression model was established to predict AIS risk. Additionally, carotid IMT and sdLDL-C were closely related to AIS severity and outcomes.

**Conclusions:**

SdLDL-C and TC were risk factors for increased IMT, while sdLDL-C was an independent risk factor. A prediction model based on IMT and other variables was established to screen the population with high AIS risk.

## Main message

Association between IMT and sdLDL-C in AIS.

## Introduction

AIS is a very common neurological disease in the world. Due to a lack of normal blood circulation, AIS leads to different degrees of ischaemia and hypoxia, resulting in malacia or necrosis of brain cells. AIS occurs mostly in elderly patients, with high rates of disability and death [1, 2]. Due to the gradual ageing of society, the incidence of cardio-cerebrovascular diseases is increasing in China [3].

Atherosclerosis is well known to contribute to the occurrence and development of ischaemic cardiovascular disease (CVD) and AIS. The pathological changes of atherosclerosis occur in the blood vessels and lead to a decrease in the diameter of the blood vessels, thus promoting the development of vascular diseases [4, 5]. IMT and carotid plaque are widely regarded as biomarkers of the severity of atherosclerosis [6, 7]. Carotid IMT has been used to detect subclinical vascular events in epidemiological studies [8], and the positive correlation of IMT and high risk of cerebro-vascular events such as stroke has been displayed in an Asian cohort [9, 10] and in a Rotterdam study [11].

Dyslipidaemia is one of the major risk factors for atherosclerosis [12]. In the previous study, AIS subjects and non-AIS people but with other neurological diseases were recruited, and the findings indicated that sdLDL-C was associated with an increased risk of AIS, especially non-cardioembolic stroke. In addition, sdLDL-C was related to AIS severity and prognosis, suggesting the importance of sdLDL-C control in patients with neurological diseases [13]. However, this study indicated that sdLDL-C was more suitable for stroke risk prediction in patients with nervous system diseases than in healthy people. It is not clear the relationship between sdLDL-C and IMT in AIS, so as to explore whether sdLDL-C plays a key role in the early stage of atherosclerosis. If it works, is it a better indicator to predict the risk of AIS in general population. One study has proven that sdLDL-C was more closely related to IMT than LDL-C and other vascular risk factors among a healthy population in China [14], but the relationship between sdLDL-C and IMT in the occurrence of AIS has not been studied.

In the current study, a positive correlation between AIS and sdLDL-C, the presence of plaque, and IMT were observed in Chinese population. SdLDL-C and TC were risk factors for increased IMT while sdLDL-C was an independent risk factor. And then sdLDL-C also contributed to unstable plaque formation. In the clinical application, a model based on IMT and sdLDL-C was useful in distinguishing high-risk AIS people in the general population.

## Methods

### Research subjects

**Fig. 1:**
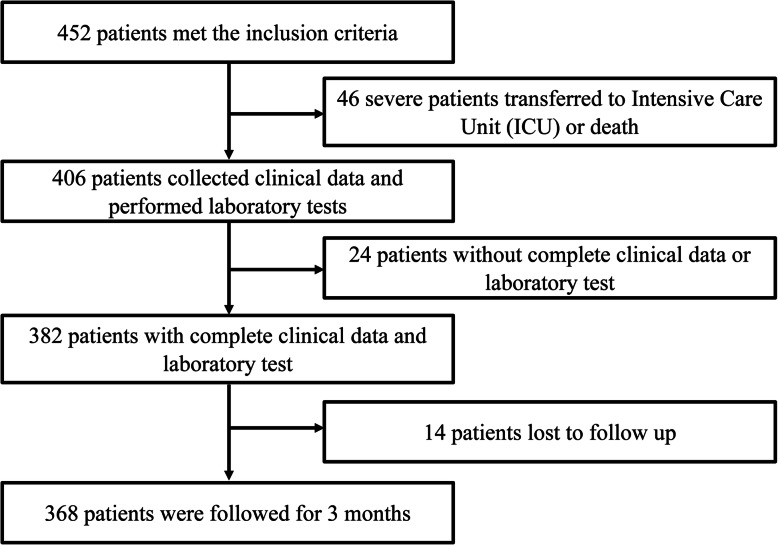
The flow chart of AIS patient’s enrolment

This is a single-centre retrospective observational study and all the procedure was approved by the Research Ethics Committee of Hubei University of Medicine. From November 2016 to February 2019, after excluding 84 patients who missed data or lost follow-up, a total of 368 consecutive patients with AIS hospitalized in the Department of Neurology, Xiangyang No. 1 People’s Hospital, Hubei Province, China, were enrolled in this study (Fig. 1). The inclusion criteria is (1) first-ever AIS, (2) a negative history of past cerebral infarction and cerebral haemorrhage, (3) a negative history of severe cardiovascular disease including congenital heart disease, myocardial infarction, rheumatic heart disease, coronary heart disease and myocarditis, (4) a negative history of severe brain pathological diseases such as primary and metastatic brain tumours (5) no transient ischaemic attack (TIA), (6) a negative history of drug abuse, and (7) loss to follow-up. One hundred sixty-five participants with no AIS were recruited as the control group in this study. The inclusion criteria were (1) a negative history of severe cardiovascular disease, (2) a negative history of severe brain pathological diseases, (3) a negative history of TIA, (4) no head trauma, (5) no signs of persistent infection. National Institutes of Health Stroke Scale (NIHSS) scores were calculated. NIHSS scores ranging from 0 to 42 represent different severities: 0 indicates no impairment, 1–4 indicates mild impairment, 5–15 indicates moderate impairment, and 16–42 indicates moderate-severe impairment. Modified Rankin Scale (mRS) was calculated to assess the outcomes [15]. Stroke subtype was grouped according to the classification of the Trial of ORG 10172 in Acute Stroke Treatment (TOAST) [16]. All-cause death is assessed as mortality at 3 months after stroke. mRS > 2 indicates dependency and mRS ≤ 2 indicates independency [17]. Smoking status was categorized as “current smokers”, indicating regular tobacco use within the last 12 months; “ex-smokers”, indicating no tobacco use within the last 12 months; and “never-smokers.” Non-smokers (current) consisted of two groups: never-smokers and ex-smokers. Current smokers were called smokers. Alcohol use status was categorized into “(current) drinker” (alcoholic beverages ≥1 per week) and “non-drinker” (those who did not drink in an average week were recorded as ‘non-drinkers’). Two physicians and one trained nurse collected the clinical information by telephone or a face-to-face interview.

### Biochemical assays

Laboratory tests were measured as soon as possible after admission, and serum lipid profiles and glucose were assessed after fasting for 8 h overnight. Serum parameters were determined by using an automatic analyser (Abbott Laboratories, Chicago, USA) based on to the manufacturer’s instructions (Supplementary Table 1). Lipoprotein subfractions were measured by using the Lipoprint LDL system (Quantimetrix Corp., CA, USA).

### Carotid artery colour Doppler ultrasound

Left and right common carotid arteries were examined through high-resolution ultrasound (Power Vision 6000; Toshiba, Tokyo, Japan). The definition of IMT was the distance between the leading edge of the media-adventitia echo and the leading edge of the internal lumen [18]. The larger IMT of the bilateral common carotid artery was the final value of the IMT. IMT > 1.0 mm was regarded as increased IMT [19]. Plaque stability was measured based on the nature of echo in ultrasound. Low-level or equal-level echo indicated unstable plaque, while high-level echo indicated stable plaque [20].

### Logistic regression model

The logistic scores to predict AIS risk probability in Chinese population were obtained according to the following equation:

AIS risk probability = $\frac{{\mathrm{e}}^{\left(\upbeta 0+\sum \upbeta \mathrm{iXi}\right)}}{1+{\mathrm{e}}^{\left(\upbeta 0+\sum \upbeta \mathrm{iXi}\right)}}$

βi indicates the coefficient of variable Xi and β0 is the constant. Xi = 1 indicating a categorical risk factor exists while 0 indicates absent. Data is displayed as area under the curve (AUC) and receiver operating characteristic (ROC) curve.

### Statistical analysis

Python software (Version 3.6) was applied to analyse the data. Categorical variables are shown as numbers combined with percentage (%). Continuous variables are shown as the mean ± standard deviation (SD). The correlation of clinical variables and IMT was analysed by Pearson correlation analysis. A logistic regression model was performed as a prediction model. Differences among groups were calculated by using the Mann-Whitney U test, the Kruskal-Wallis H test combined with the chi-square test. Statistically significant differences were *P* <  0.05.

## Results

### Group comparisons

The median IMT of the patients and controls was 1.09 ± 0.10 mm and 1.06 ± 0.10 mm, respectively (Supplementary Figure 1). Carotid plaque was detected in 303 AIS patients (82.34%) and 117 controls (70.91%). A higher prevalence of hypertension was shown in the AIS group than in the controls (72.28% vs. 59.39%). The levels of glucose, TG and sdLDL-C (LDL3–7 -C) in the AIS group were higher compared to the controls (Table 1). A positive correlation between AIS and sdLDL-C (*r* = 0.29, *P* <  0.001), the presence of plaque (*r* = 0.21, *P* <  0.001), glucose (*r* = 0.14, *P* = 0.001), IMT thickness (*r* = 0.13, *P* = 0.002) and presence of hypertension (*r* = 0.12, *P* = 0.003) was observed via Pearson correlation analysis (Supplementary Figure 2).

**Table 1:** Baseline characteristics of patients and controls

Clinical variables	AIS patients (*n* = 368)	Controls (*n* = 165)	*P* value
Age (years)^*^	63.58 ± 11.71	63.02 ± 11.84	0.273
Male/Female sex	216 (58.70)/152 (41.30)	96 (58.18)/69 (41.81)	0.987
BMI (kg/m^2^)^*^	23.55 ± 2.80	23.58 ± 2.64	0.213
NIHSS	6.28 (0–33)	/	/
mRS	1.06 (0–5)	/	/
IMT (mm)^*^	1.09 ± 0.10	1.06 ± 0.10	0.007
Carotid plaques	303 (82.34)	117 (70.91)	0.004
Hypertension	266 (72.28)	98 (59.39)	0.004
Diabetes	97 (26.36)	33 (20.00)	0.141
Smoker	109 (29.62)	47 (28.48)	0.870
Drinker	95 (25.82)	40 (24.24)	0.781
Glucose (mmol/L)^*^	6.71 ± 2.27	6.06 ± 1.68	< 0.0001
TC (mmol/L)^*^	3.85 ± 0.91	3.71 ± 0.86	0.102
TG (mmol/L)^*^	1.42 ± 1.08	1.28 ± 0.69	0.021
LDL-C (mmol/L)^*^	2.18 ± 0.65	2.16 ± 24	0.328
sdLDL-C (mg/dl)^*^	24.44 ± 20.40	13.17 ± 6.01	< 0.0001
HDL-C (mg/dl)^*^	41.91 ± 12.70	42.83 ± 11.01	0.163
Non-HDL-C (mmol/L)^*^	24.32 ± 20.28	23.19 ± 10.20	0.248
Statin therapy	15 (4.10)	10 (6.06)	0.435

Continuous variables are shown as the mean ± standard deviation (SD) marked with an asterisk (*). Categorical variables are shown as numbers combined with percentage (%).NIHSS and mRS are presented as the mean (minimal and maximal values). *BMI* body mass index, *NIHSS* National Institutes of Health Stroke Scale, *mRS* modified Rankin Scale, *IMT* intima-media thickness, *TC* total cholesterol, *TG* triglycerides, *LDL-C* low-density lipoprotein cholesterol, *sdLDL-C* small dense low-density lipoprotein cholesterol, *HDL-C* high-density lipoprotein cholesterol

### Relationship of IMT with other clinical factors

According to the cut-off thickness of 1.0 mm, 60.33% of patients had increased IMT. The level of sdLDL-C was markedly higher in the IMT thickening group than that in the normal IMT group (Table 2).

**Table 2:** Clinical features of all participants in the normal and increased IMT groups

Variable	Normal (IMT ≤ 1.0 mm) (*n* = 146)	Thickened (IMT > 1.0 mm) (*n* = 222)	*P* value
Age (years)^*^	64.56 ± 11.33	62.93 ± 11.93	0.115
Male/Female sex	91 (62.33)/55 (37.67)	125 (56.31)/97 (43.69)	0.298
BMI (kg/m^2^)^*^	23.48 ± 2.76	23.59 ± 2.84	0.202
IMT (mm)^*^	0.98 ± 0.05	1.17 ± 0.09	< 0.0001
Carotid plaques	119 (81.51)	184 (82.29)	0.840
Hypertension	101 (69.18)	165 (74.34)	0.337
Diabetes	41 (28.08)	56 (25.23)	0.626
Smoker	42 (28.77)	67 (30.18)	0.862
Drinker	34 (23.29)	61 (27.48)	0.437
Glucose (mmol/L)^*^	6.78 ± 2.62	6.67 ± 2.01	0.105
TC (mmol/L)^*^	3.76 ± 0.88	3.90 ± 0.93	0.066
TG (mmol/L)^*^	1.51 ± 1.44	1.36 ± 0.76	0.367
LDL-C (mmol/L)^*^	2.16 ± 0.65	2.20 ± 0.65	0.187
sdLDL-C (mg/dl)^*^	21.97 ± 20.29	26.07 ± 20.37	0.008
HDL-C (mg/dl)^*^	41.76 ± 12.02	42.01 ± 13.15	0.377
Non-HDL-C (mmol/L)^*^	24.52 ± 27.36	24.19 ± 13.86	0.130
Statin therapy	6 (4.11%)	9 (4.05%)	0.808

Continuous variables are shown as the mean ± standard deviation marked with an asterisk (*). Categorical variables are shown as numbers combined with percentage (%)

Chi-square test for discrete values and Mann-Whitney U test for continuous values

### Association between carotid plaque and other clinical factors

The levels of sdLDL-C, LDL-C and TC were highest in patients with unstable plaques and lowest in subjects without plaques. People with unstable carotid plaque had the highest IMT (*P* <  0.0001) (Table 3).

**Table 3:** Characteristics of participants in the carotid plaque-free group and plaque group

Variable	No Plaque (*n* = 65)	Stable Plaque (*n* = 199)	Unstable Plaque (*n* = 104)	*P* value
Age (years)^*^	59.86 ± 13.14	65.23 ± 9.86	62.75 ± 13.41	0.009
Male/Female sex	42 (64.62)/23 (35.38)	123 (61.81)/76 (38.19)	51 (49.04)/53 (50.96)	0.057
BMI (kg/m^2^)^*^	23.78 ± 3.20	23.47 ± 2.71	23.54 ± 2.74	0.741
IMT (mm)^*^	1.07 ± 0.10	1.06 ± 0.09	1.18 ± 0.10	< 0.0001
Hypertension	48 (73.84)	142 (71.36)	76 (73.08)	0.906
Diabetes	14 (24.54)	55 (27.64)	28 (26.92)	0.617
Smoker	20 (30.77)	64 (32.16)	25 (24.04)	0.331
Drinker	13 (20.00)	56 (28.14)	26 (25.00)	0.417
Glucose (mmol/L)^*^	6.54 ± 2.08	6.73 ± 2.48	6.79 ± 1.97	0.210
TC (mmol/L)^*^	3.49 ± 0.71	3.82 ± 0.92	4.12 ± 0.93	< 0.0001
TG (mmol/L)^*^	1.35 ± 0.76	1.44 ± 1.33	1.43 ± 0.65	0.050
LDL-C (mmol/L)^*^	1.89 ± 0.48	2.19 ± 0.63	2.44 ± 0.70	< 0.0001
sdLDL-C (mg/dl)^*^	14.78 ± 10.64	21.30 ± 18.15	36.50 ± 23.56	< 0.0001
HDL-C (mg/dl)^*^	40.52 ± 12.95	43.16 ± 13.07	40.39 ± 11.63	0.234
Non-HDL-C (mmol/L)^*^	28.07 ± 37.70	23.37 ± 15.18	23.79 ± 11.36	0.338
Statin therapy	1 (1.54)	12 (6.03)	2 (1.92)	0.120

Continuous variables are shown as the mean ± standard deviation (SD) marked with an asterisk (*). Categorical variables are shown as numbers combined with percentage (%)

Chi-square test for discrete values and Kruskal-Wallis H test for continuous values

### SdLDL-C was an independent risk factor for IMT

**Fig. 2:**
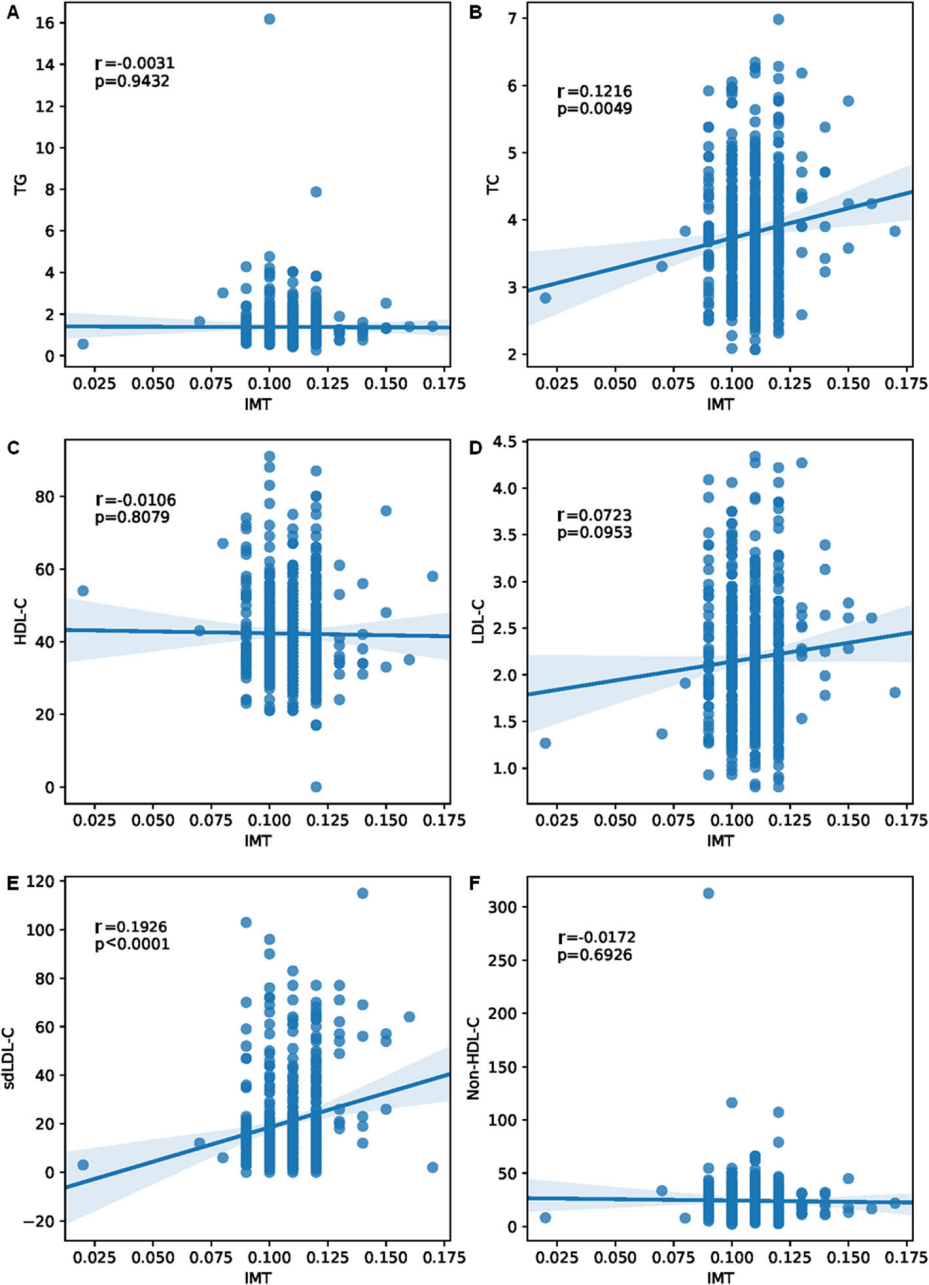
Pearson correlation analysis of IMT and other clinical factors

A significant and positive correlation between the presence of carotid plaques and IMT in the AIS cohort was observed (*r* > 0.1, *P* <  0.05) (Supplementary Table 2). IMT was positively correlated with the levels of sdLDL-C and TC among various lipid parameters (*r* = 0.19 and 0.12, respectively, *P* <  0.05) (Fig. 2). When recalculating the odds ratio (OR) value in the multivariable logistic regression model, it was found that two variables with OR > 1 and *P* <  0.05, TC and sdLDL-C, were two risk factors for increased IMT (Supplementary Table 3). SdLDL-C was a risk factor for increased IMT in the basic model (base model) (β, 0.018; OR, 1.03; 95% confidence interval (CI), 1.005–1.031; *P* = 0.005) and an independent risk factor in the model 1 (β, 0.016; OR, 1.03; 95% CI, 1.003–1.029; *P* = 0.017) after additional adjustment for age, gender, the presence of diabetes mellitus, SBP, DBP, smoking, alcohol consumption and body mass index (BMI) (Table 4 and Supplementary Table 4).

**Table 4:** Multivariate logistic regression analysis of the independent correlation between sdLDL-C and IMT

	β	SE	Wald χ^2^	OR value	95.0% CI for OR	*P*
Base model	0.018	0.006	2.783	1.03	1.005–1.031	0.005
Model 1	0.016	0.007	2.391	1.03	1.003–1.029	0.017

*OR* odds ratio

*CI* confidence interval

Model 1 additionally adjusted for gender, age, SBP, DBP, diabetes mellitus, smoking, drinking and BMI

### A prediction model for AIS risk based on IMT and other vascular risk factors

**Fig. 3:**
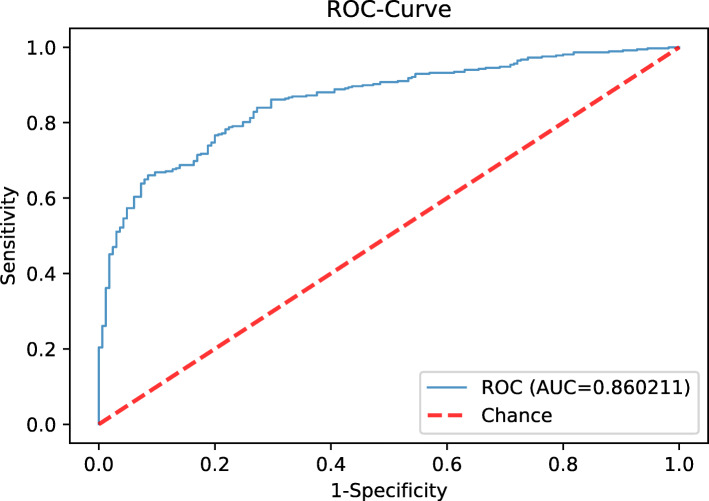
A prediction model for AIS risk in general population

ROC curves were plotted the for IMT and serum lipid biomarkers to discriminate AIS patients from the controls (Supplementary Figure 3). The AUC of IMT for AIS was 0.564, which was lower than that of sdLDL-C (0.644) but higher than those of LDL-C (0.506) and non-HDL-C (0.491). The predictive value of IMT for AIS was better than those of non-HDL-C and LDL-C but not as good as that of sdLDL-C. A logistic regression model based on comprehensive factors including IMT and sdLDL-C for assessing the risk of AIS was built (AUC = 0.86) (Fig. 3). Conventional risk factors: age, BMI, smoking, SBP, DBP, glucose, IMT, carotid plaque, sdLDL-C, LDL-C, LDL1-C, LDL2-C, TC, TG and HDL-C were adapted to the model.

### Association between sdLDL-C, IMT and AIS severity and prognosis

No significant difference in IMT or carotid plaques was found in the subtypes of AIS. A positive correlation of AIS severity with IMT was observed, while sdLDL-C was different between the mild impairment group and no impairment group (*P* <  0.05) (Supplementary Table 5). Carotid IMT and sdLDL-C were related to poor outcomes (Supplementary Table 6).

## Discussion

Cardiovascular and cerebrovascular diseases have the highest morbidity and mortality in the clinical setting. AIS is the most common cerebrovascular disease in the world [21]. AIS is caused by carotid stenosis or obstruction due to atherosclerotic plaque formation, rupture and erosion [22]. Because carotid IMT is correlated to the risk and development of ischemic stroke [23]. IMT has been used as an indicator of stroke in several observational and clinical trials in different populations [11, 24]. A positive correlation of IMT and the incidence of stroke among young people suggested the role of IMT in all age groups [5]. In this study, it was found that the mean IMT was markedly increased in the AIS population compared with that in the controls, which is in agreement with the abovementioned findings. IMT significantly increased in the unstable plaque group. In addition, correlation analysis revealed that IMT was positively associated with the presence of carotid plaque, indicating that with the higher the IMT, more unstable plaque exists, and then the more severe AIS will be.

When paying attention to the impact of IMT and plaque on AIS, it is must realize that the pathological change in atherosclerosis leads to the reduction in blood vessel diameter due to the hypertrophy or entropy change in blood vessels, which acts as the fundamental cause of the promotion of cerebrovascular diseases development. Because many cerebrovascular diseases occur suddenly without any clinical symptoms, it is essential to prevent the diseases at the early stage of atherosclerosis. To detect early vascular wall damage, which would play a vital role in the atherosclerosis progression, risk factors for increased carotid IMT should be identified.

Hypertension, hyperglycaemia and dyslipidaemia were confirmed to be related to IMT [25]. Among these conditions, dyslipidaemia was one of the most fundamental risk factors. Previous reports have proven that TC, TG, and LDL-C were associated with AIS [26]. Additionally, the LDL-C level has been shown to be a useful biomarker to predict stroke susceptibility. The pivotal role of LDL-C in the development of atherosclerosis has been widely accepted [27]. Recently, studies have confirmed that a portion of the population with atherosclerosis or stroke has normal LDL-C [28]. Thus, current research began to focus on exploring better lipid biomarkers for atherosclerosis. LDL-C, consisting of particles with different densities, sizes and chemical properties [29], is classified into 2 types: lbLDL-C (LDL-C 1–2) and sdLDL-C (LDL-C 3–7, small and dense LDL-C). An increasing number of studies have demonstrated that sdLDL-C is closely correlated with carotid plaques formation and atherosclerosis [30, 31]. Previous study indicated that sdLDL-C was related to an increased risk of AIS, especially noncardioembolic stroke, in patients with neurological diseases. In addition, sdLDL-C was related to AIS severity and outcomes, suggesting the importance of sdLDL-C in distinguishing stroke from other neurological diseases [13].

Because many cerebrovascular diseases occur suddenly with no clinical symptoms, preventing diseases in the early stage of atherosclerosis will be an effective approach. Hence, the main objective of this study is to determine risk factors, including sdLDL-C, LDL-C, TC, etc., for increased IMT because dyslipidaemia acts as a critical risk factor for atherosclerosis [12]. Previous researches demonstrated a close relationship between IMT and sdLDL-C in healthy men at the age of 50 years and in subjects with dyslipidaemia [32, 33]. In the current study, the level of sdLDL-C elevated more in the increased IMT cohort than that in the normal IMT cohort. Multivariate logistic regression analysis indicated that sdLDL-C and TC were risk factors for increased IMT, while sdLDL-C became an independent predictor of elevated IMT.

AIS is associated with plaques properties, so plaques stability or not is important to the occurrence and development of AIS. In this study, sdLDL-C level was highest in patients with unstable plaques and lowest in subjects without plaques, suggesting that sdLDL-C may contribute to unstable plaque. Another study showed that the level of sdLDL-C was markedly higher in the unstable plaque people than that in the stable plaque as well as non-plaque group. Furthermore, sdLDL-C was proved to be an independent risk factor for unstable plaques by using the logistic regression analysis [34]. The possible mechanism may be that the characteristics of unstable plaques such as concomitant macrophage aggregation and decreased smooth muscle cell proliferation are related to denser LDL subfractions, for example, sdLDL-C. The denser the LDL, the easier it is to reach the subendothelial layer where they are more likely to be oxidized [35]. In addition, denser LDL particles have a higher affinity for arterial proteoglycans, thus prolonging the residence time in the arterial wall, and inducing the development of atherosclerosis [36]. Statins, fibrates, ezetimibe and nicotinic acid etc. are the common drugs to reduce sdLDL-C in the treatment of dyslipidemia. In dietary intervention, food containing avocado, pistachio and soybean are recommended to decrease the sdLDL-C level. This study suggested that combined with the traditional lipid risk factors, sdLDL-C should be considered as a priority parameter to screen and a better target to lower lipid in vivo [37].

In addition, results showed that sdLDL-C could be used with IMT and other factors to predict AIS risk. The predictive significance of single factors, such as IMT, LDL-C and even sdLDL-C, is not ideal, so a prediction model for AIS risk was built. This model was better than the previously established model that is used to screen high-risk individuals among patients with neurological diseases (AUC = 0.86 vs. AUC = 0.81). Compared with the previous model, this model not only increases variables such as age, BMI, SBP, DBP, smoking, IMT and plaque but can also be applied to a wider range of individuals, which is of great significance for the detection of early asymptomatic atherosclerosis and the prevention of cerebrovascular events in Chinese adults. Because AIS is associated with a combination of multiple risk factors, multiple risk factors together could predict the risk more effectively than single risk factors such as IMT and sdLDL-C. This study suggested that comprehensive factors, including IMT, sdLDL-C and other variables related to the carotid artery, can reflect the vascular state more comprehensively and play a vital and objective role in the occurrence, development and prognostic prediction of ischaemic stroke.

Consistent with previous studies, IMT and sdLDL-C values were positively associated with AIS severity. These correlations were higher than those of other vascular risk factors [38–40]. In addition to lipid risk factors, other risk factors, such as IMT and carotid plaque, were identified as meaningful clinical biomarkers for AIS outcomes.

### Study strengths and limitations

This is the first report demonstrating that sdLDL-C is an independent risk factor for increased IMT after additional adjustment for gender, BMI, age and other traditional risk factors, indicating the role of sdLDL-C in the early stage of atherosclerosis. A prediction model based on IMT and sdLDL-C was established to screen high AIS risk in the general population. However, there are some limitations in the current study listed as follows. First, the current study is a retrospective study, and the size of the participants enrolled was relatively small. Because of the nature of the research design, only relevant conclusions can be drawn without mechanistic research. Second, this study involved individuals in the Han population in the same region. Therefore, future research needs to expand the sample size. Third, in this study, only the IMT and carotid plaque data detected by ultrasound were collected and analysed. Fourth, the effects of inflammatory markers on atherosclerosis were not considered in this study. In addition, since sex hormones are not routinely tests in the hospital, the estrogen and androgen levels were not detected in this study. Sex hormones should be considered in the future study. The correlations between ischaemic stroke and quantitative indicators, such as the number of carotid plaques and plaque thickness and area, need further research.

## Conclusion

Increased IMT is a marker to detect early vascular wall damage, which has important contribution to the occurrence and development of atherosclerosis. Here, results showed that sdLDL-C and TC were risk factors for IMT, while sdLDL-C was an independent risk factor. One prediction model based on IMT and other variables for AIS risk was constructed to help clinicians better prevent AIS. Thus, controlling sdLDL-C levels is of great significance for maintaining normal IMT, reducing plaque formation and preventing atherosclerosis and stroke in the early stage.

## Supplementary information

**Additional file 1: Figure S1.** Scatter plot of IMT in the AIS and control group. **Figure S2.** Pearson correlation analysis of clinical risk factors and AIS risk. **Figure S3.** Predictive values of IMT and other lipid parameters for AIS risk. Areas under the curves: 0.564 for IMT, 0.644 for sdLDL-C, 0.506 for LDL-C, 0.491 for non-HDL-C. **Supplementary Table 1.** Details of reagents used in the automatic biochemical analyzer**. Supplementary Table 2.** Spearman correlations analysis of carotid IMT and clinical variables. **Supplementary Table 3.** Multivariable logistic regression analysis of glucose and lipid risk factors for IMT. **Supplementary Table 4.** Multivariable logistic regression analysis of clinical risk factors for IMT. **Supplementary Table 5.** Association between AIS severity and vascular risk factors. **Supplementary Table 6.** Association between AIS outcomes and vascular risk factors.

## Data Availability

All data are contained in the published article and supplementary materials.

## Abbreviations

LDL-C: Low-density lipoprotein cholesterol
BMI: Body mass index
AIS: Acute ischaemic stroke
IMT: Intima-media thickness
sdLDL-C: small dense low-density lipoprotein cholesterol
TG: Triglycerides
TC: Total cholesterol
HDL-C: High-density lipoprotein cholesterol
ROC: Receiver operating characteristic
AUC: Area under the curve
