# Supplementary material for: Association between carotid intima media thickness and small dense low-density lipoprotein cholesterol in acute ischaemic stroke
Source: Lipids Health Dis. 2020 Jul 28;19:177. doi: 10.1186/s12944-020-01353-0 (PMC7388515; doi:10.1186/s12944-020-01353-0)
Supplement: Supplementary file 1 — Additional file 1: Figure S1. Scatter plot of IMT in the AIS and control group. Figure S2. Pearson correlation analysis of clinical risk factors and AIS risk. Figure S3. Predictive values of IMT and other lipid parameters for AIS risk. Areas under the curves: 0.564 for IMT, 0.644 for sdLDL-C, 0.506 for LDL-C, 0.491 for non-HDL-C. Supplementary Table 1. Details of reagents used in the automatic biochemical analyzer. Supplementary Table 2. Spearman correlations analysis of carotid IMT and clinical variables. Supplementary Table 3. Multivariable logistic regression analysis of glucose and lipid risk factors for IMT. Supplementary Table 4. Multivariable logistic regression analysis of clinical risk factors for IMT. Supplementary Table 5. Association between AIS severity and vascular risk factors. Supplementary Table 6. Association between AIS outcomes and vascular risk factors. [file 12944_2020_1353_MOESM1_ESM.docx]

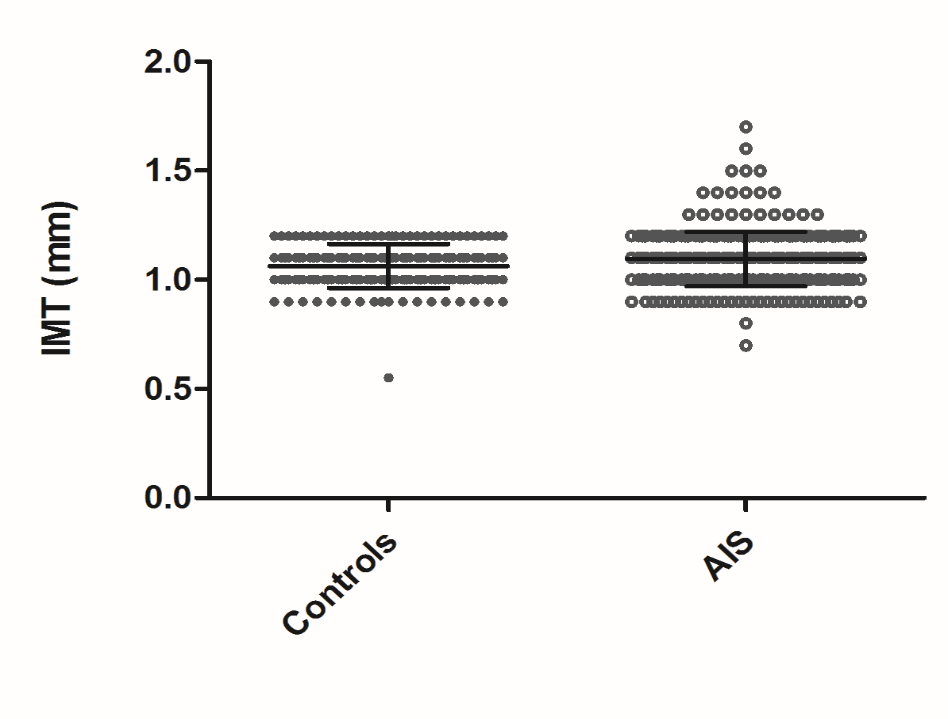


Fig S1. Scatter plot of IMT in the AIS and control group


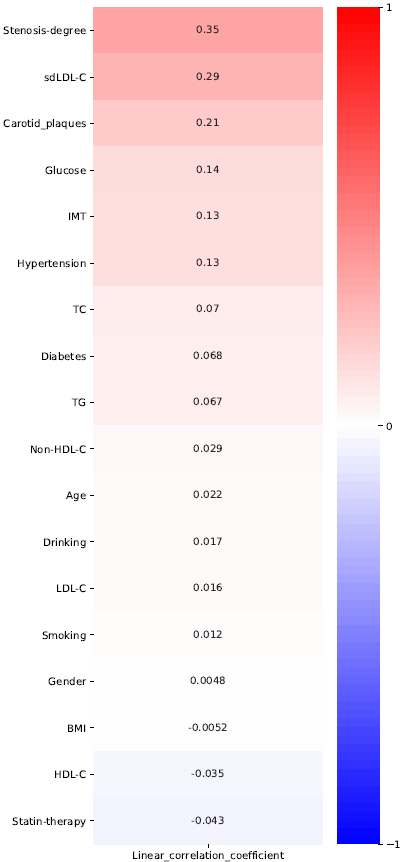


Fig S2. Pearson correlation analysis of clinical risk factors and AIS risk.

Fig S3. Predictive values of IMT and other lipid parameters for AIS risk

Areas under the curves: 0.564 for IMT, 0.644 for sdLDL-C, 0.506 for LDL-C, 0.491 for non-HDL-C.

Supplementary Table 1. Details of reagents used in the automatic biochemical analyzer

| Item | Manufacturer | Reagent | Lot number | Quality control reagent | Calibration reagent |
| --- | --- | --- | --- | --- | --- |
| Glucose | Biosino Bio-Technology and Science Inc. Beijing, China | Glucose test kit | 92609073 | Bio-Rad Laboratories, Inc. California, USA | Biosino Bio-Technology and Science Inc. Beijing, China |
| TC | Abbott Laboratories. Chicago, USA | Cholesterol test kit | 48698UN19 | Bio-Rad Laboratories, Inc. California, USA | Abbott Laboratories. Chicago, USA |
| TG | Abbott Laboratories. Chicago, USA | Triglycerides test kit | 72243UN19 | Bio-Rad Laboratories, Inc. California, USA | Abbott Laboratories. Chicago, USA |
| LDL-C | Biosino Bio-Technology and Science Inc. Beijing, China | Low density lipoprotein cholesterol test kit | 92650122 | Bio-Rad Laboratories, Inc. California, USA | Biosino Bio-Technology and Science Inc. Beijing, China |
| HDL-C | Biosino Bio-Technology and Science Inc. Beijing, China | High density lipoprotein cholesterol test kit | 92649105 | Bio-Rad Laboratories, Inc. California, USA | Biosino Bio-Technology and Science Inc. Beijing, China |

Supplementary Table 2. Spearman correlations analysis of carotid IMT and clinical variables

| Variable | r | *P* value |
| --- | --- | --- |
| Age (years) | -0.105 | 0.653 |
| Male / Female sex | -0.023 | 0.050 |
| BMI (kg/m^2^) | 0.018 | 0.735 |
| Carotid plaques | 0.232 | < 0.0001 |
| Hypertension | 0.024 | 0.641 |
| Diabetes | -0.022 | 0.669 |
| Smoker | 0.008 | 0.873 |
| Drinker | 0.008 | 0.873 |
| Glucose | 0.075 | 0.150 |
| Statin therapy | 0.027 | 0.603 |

Supplementary Table 3. Multivariable logistic regression analysis of glucose and lipid risk factors for IMT

| Factors | B-value | SE | Walsχ2 | OR value | 95.0% CI for OR | *P* |
| --- | --- | --- | --- | --- | --- | --- |
| SdLDL-C | 0.018 | 0.006 | 2.783 | 1.03 | 1.005-1.031 | 0.005 |
| TC | 1.722 | 0.542 | 3.176 | 5.59 | 1.933-16.184 | 0.001 |
| TG | -0.253 | 0.196 | -1.292 | 0.78 | 0.529-1.140 | 0.196 |
| LDL-C | -1.792 | 0.567 | -3.159 | 0.17 | 0.055-0.507 | 0.002 |
| HDL-C | -0.033 | 0.016 | -2.078 | 0.97 | 0.937-0.998 | 0.038 |
| Non-HDL-C | -0.032 | 0.017 | -1.856 | 0.97 | 0.936-1.002 | 0.063 |
| Glucose | 0.049 | 0.047 | 1.040 | 1.05 | 0.958-1.150 | 0.298 |

OR, odds ratio; CI, confidence interval.

Supplementary Table 4. Multivariable logistic regression analysis of clinical risk factors for IMT

| Factors | B-value | SE | Walsχ2 | OR value | 95.0% CI for OR | *P* |
| --- | --- | --- | --- | --- | --- | --- |
| SdLDL-C | 0.016 | 0.007 | 2.391 | 1.03 | 1.003-1.029 | 0.017 |
| TC | 1.058 | 0.354 | 2.988 | 2.88 | 1.439-5.760 | 0.003 |
| TG | -0.387 | 0.183 | -2.110 | 0.68 | 0.474-0.973 | 0.035 |
| LDL-C | -1.187 | 0.411 | -2.890 | 0.31 | 0.136-0.682 | 0.004 |
| HDL-C | -0.018 | 0.012 | -1.521 | 0.98 | 0.961-1.005 | 0.128 |
| Glucose | 0.046 | 0.055 | 0.832 | 1.05 | 0.940-1.168 | 0.405 |
| Age | 0.002 | 0.009 | 0.218 | 1.00 | 0.985-1.019 | 0.828 |
| Gender | -0.489 | 0.236 | -2.070 | 0.61 | 0.386-0.974 | 0.059 |
| BMI | 0.034 | 0.036 | 0.947 | 1.03 | 0.965-1.110 | 0.343 |
| SBP | 0.020 | 0.011 | 1.892 | 1.02 | 0.999-1.042 | 0.059 |
| DBP | -0.013 | 0.016 | -0.809 | 0.99 | 0.956-1.019 | 0.418 |
| Diabetes | -0.116 | 0.277 | -0.418 | 0.89 | 0.518-1.533 | 0.676 |
| Smoking | 0.214 | 0.348 | 0.613 | 1.24 | 0.626-2.452 | 0.540 |
| Drinking | -0.026 | 0.352 | -0.073 | 0.97 | 0.489-1.941 | 0.942 |

OR, odds ratio; CI, confidence interval.

Supplementary Table 5. Association between AIS severity and vascular risk factors

| Clinical factors | None(n=39) | Mild (n=158) | Moderate-severe (n=113) | Severe (n=58) |
| --- | --- | --- | --- | --- |
| IMT (mm)^*^ | 1.06±0.09^a^ | 1.07±0.10^a^ | 1.11±0.10^b^ | 1.14±0.20^b^ |
| Carotid plaques | 24(61.54)^a^ | 131(82.91)^b^ | 93(82.30)^b^ | 55(94.83)^c^ |
| sdLDL-C (mg/dl)^*^ | 8.87±4.19^a^ | 12.35±6.21^b^ | 27.07±11.50^c^ | 62.74±15.01^d^ |
| LDL-C (mmol/L)^*^ | 1.87±0.51^a^ | 1.94±0.54^a^ | 2.36±0.65^b^ | 2.70±0.61^c^ |
| TC (mmol/L)^*^ | 3.33±0.58^a^ | 3.59±0.77^a^ | 4.07±0.96^b^ | 4.45±0.89^c^ |

Categorical variables are presented as numbers and proportions (%), and continuous variables are presented as the mean ± standard deviation (SD) ^*^.

Groups that are not labeled with any of the same letters (^a^, ^b^, ^c^) indicate statistically significant at 0.05. Mann-Whitney U test for continuous values and chi-square test for discrete values.

Supplementary Table 6. Association between AIS outcomes and vascular risk factors

| Clinical factors | Mortality (n= 6) | Dependency (n= 50) | Independency (n= 312) | *P* value |
| --- | --- | --- | --- | --- |
| NIHSS^*^ | 17.00±6.90 | 15.98±756 | 4.52±4.70 | < 0.0001 |
| IMT (mm)^*^ | 1.23±0.20 | 1.14±0.20 | 1.08±0.10 | 0.005 |
| Carotid plaques | 6(100.00) | 47(94.00) | 250(80.13) | < 0.0001 |
| sdLDL-C (mg/dl)^*^ | 64.17±35.87 | 50.22±23.08 | 19.55±15.02 | < 0.0001 |
| LDL-C (mmol/L)^*^ | 2.58±0.67 | 2.65±0.73 | 2.10±0.61 | < 0.0001 |
| TC (mmol/L)^*^ | 4.42±0.86 | 4.43±1.05 | 3.74±0.85 | < 0.0001 |
| TG (mmol/L)^*^ | 1.26±0.35 | 1.59±0.81 | 1.40±1.13 | < 0.0001 |

Categorical variables are presented as numbers and proportions (%), and continuous variables are presented as the mean ± standard deviation (SD) ^*^.

Mann-Whitney U test for continuous values and chi-square test for discrete values.
